# Semiflexible Biopolymers in Bundled Arrangements

**DOI:** 10.3390/polym8080274

**Published:** 2016-07-28

**Authors:** Jörg Schnauß, Tina Händler, Josef A. Käs

**Affiliations:** 1 Institute for Experimental Physics I, Universität Leipzig, Linnéstraße 5, Leipzig 04103, Germany; tina.haendler@uni-leipzig.de (T.H.); jkaes@physik.uni-leipzig.de (J.A.K.); 2 Fraunhofer Institute for Cell Therapy and Immunology, Perlickstraße 1, Leipzig 04103, Germany

**Keywords:** semiflexible polymers, bundles, worm-like bundle, dynamics, actin, microtubules, depletion forces, counterion condensation, crosslinkers

## Abstract

Bundles and networks of semiflexible biopolymers are key elements in cells, lending them mechanical integrity while also enabling dynamic functions. Networks have been the subject of many studies, revealing a variety of fundamental characteristics often determined via bulk measurements. Although bundles are equally important in biological systems, they have garnered much less scientific attention since they have to be probed on the mesoscopic scale. Here, we review theoretical as well as experimental approaches, which mainly employ the naturally occurring biopolymer actin, to highlight the principles behind these structures on the single bundle level.

## 1. Introduction

Semiflexible polymers cannot be treated in the classical physical frame of flexible polymers or rigid rods due to their non-vanishing backbone stiffness. While they still show strong thermal fluctuations, they remain in an outstretched configuration, which lends them unique mechanical properties. During the last few decades, the worm-like chain (WLC) model was established as the standard description for this special class of polymers. Its defining property is the bending stiffness κ as an intrinsic material constant [1]. Many experimental studies have successfully demonstrated the applicability of this description to a broad range of biopolymers, which are mainly considered to be semiflexible. From a biological point of view, the unique mechanical properties of this polymer class allow the formation of stable networks even for low volume fractions, which provides biological material with mechanical stability while still enabling transport processes [2]. These network arrangements can be modeled via collective properties of WLCs. Within entangled networks, the WLC model even allows derivations of the scaling of the elastic plateau shear modulus with regard to polymer concentration and their according single filament stiffness [3,4,5]. Due to their biological importance and experimental accessibility, networks received a lot of scientific attention [6,7,8,9].

In contrast, the arrangement of WLCs into bundled structures received much less attention, although they play key roles in biological matter. A natural model system for semiflexible polymers is the protein actin, which can polymerize into long, semiflexible filaments. Besides forming isotropic networks, they can be assembled into bundles via a variety of effects, and filaments are either parallel or antiparallel aligned. Thus, single bundles are anisotropic singularities in a system impeding bulk measurements such as shear rheology, which commonly relies on an isotropic, homogenous material distribution throughout the entire sample. Anisotropies do not average out and highly bias each experiment in a different way rendering controlled investigations challenging (except networks of bundles spanning the entire sample [10,11,12]). Each bundle needs to be experimentally probed on the mesoscopic scale and has to be investigated separately since bundles do not form uniformly. Actin filaments, for instance, have a polydisperse length distribution leading to length and thickness variations of the overall structure. Despite those limitations, biopolymers are a suitable model system to study bundles of semiflexible polymers since they are readily experimentally accessible and can be investigated via a multitude of experimental methods such as fluorescence microscopy [13,14,15], optical tweezers [16,17], atomic force microscopy [18,19,20,21], light scattering [22,23], electron microscopy [14,24,25,26], and X-ray diffraction [27,28]. Throughout this review, we mainly focus on the protein actin as one of the most abundant biological components but also emphasize findings for other biopolymers such as microtubules to illustrate mutual, rather general concepts.

Studies on single bundles require sophisticated experiments to uncover their physical principles to gain a deeper understanding of their crucial cellular functions. In biological systems, for instance, bundles are mainly formed by crosslinking proteins, which add further parameters to the WLC model and introduce their own inherent mechanical contributions. First mathematical descriptions incorporate these material properties for distinct boundary conditions of the bundle within the frame of the worm-like bundle model (WLB) [29,30].

To determine contributions of crosslinkers, in vitro experiments of single bundles formed without any crosslinkers are inevitable. Commonly employed bundling mechanisms are summarized and arising effects without crosslinking components are highlighted and explained within according theoretical approaches. Subsequently, the large impact of crosslinkers is reviewed from an experimental as well as theoretical perspective to illustrate their influences to mechanical as well as dynamical properties of bundled structures. For the interested reader, we also recommend a related review by Benetatos and Jho [31], which focuses on recent theoretical and computational advances from a different perspective.

## 2. Bundle Formation

**Figure 1:**
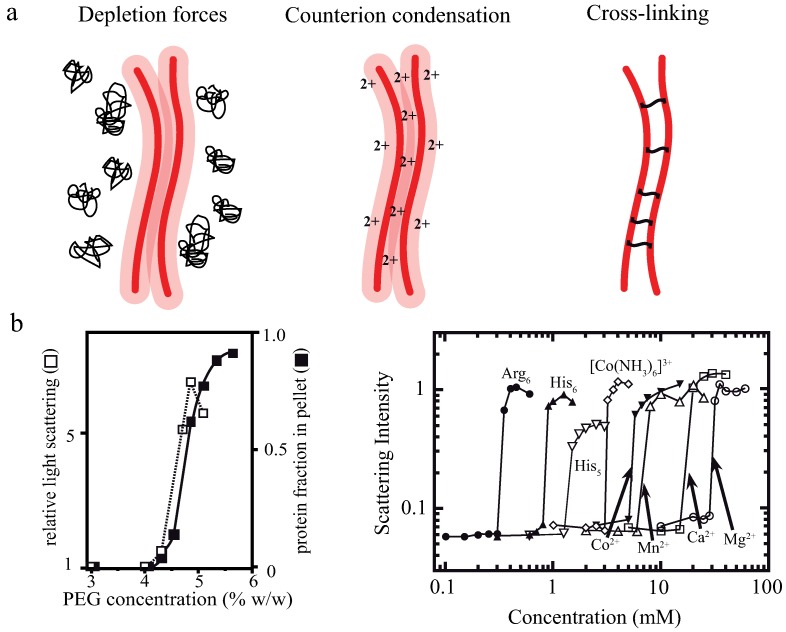
(**a**) Shown are three bundling mechanisms for actin filaments used for experiments described within this review. Actin filaments can be either bundled by fundamental physical effects such as depletion forces (e.g., induced by polyethylene glycol (PEG)) or counterion condensation but also by additional crosslinking proteins; (**b**) light scattering experiments revealed specific threshold concentrations and displayed rather sharp transitions from isotropically distributed filaments to ordered bundles when depletion forces or counterion condensation were employed in a concentration dependent manner. The graphs are adapted from Suzuki et al. as well as from J.X. Tang and P.A. Janmey [22,23].

Actin filaments are negatively charged along their surface. To achieve bundling of these filaments, attractions are needed to overcome arising electrostatic repulsions. An overview of three potential bundling mechanisms is given in Figure 1 displaying two fundamental physical principles—depletion forces and counterion condensation—which counteract the repulsive forces. The third mechanism employs additional, crosslinking components connecting two or more filaments as found in biological systems. In cells, these crosslinking proteins usually undergo binding and unbinding events with different on- and off- rates for differing crosslinkers [2,32,33].

The addition of crosslinking proteins significantly alters a bundle’s properties. Their features contribute to the mechanical behavior of the overall structure as described within the WLB theory. To investigate bundles without additional contributions, it is necessary to arrange actin filaments without accessory proteins. A first possibility is bundle formation via depletion forces. Due to molecular crowding, filaments experience an attracting force leading to an overlap of their excluded volumes. Within this process, the free energy of the system is decreased and entropy increased [34,35,36]. A second approach employs high concentrations of positive, divalent ions in solution to cause bundling via counterion condensation, where divalent counterions can compensate the partial surface charges of two filaments establishing an ion-based crosslinker [23].

The interplay of the rich cellular protein pool and fundamental physical principles renders in vivo studies of actin bundles difficult. To determine their mechanical properties and to distinguish between contributions of involved components, in vitro investigations are inevitable. The potential to form bundles without crosslinkers allows for investigation of their mechanical properties decoupled from contributions of additional components.

### 2.1. Depletion Interaction

**Figure 2:**
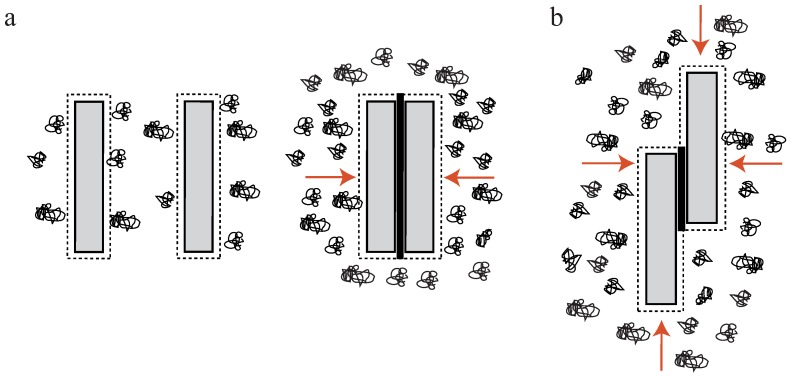
(**a**) High concentrations of non-interacting polymers—so-called crowding agents—in solution are causing attractive forces between colloidal, rod-like particles [35,36] when exceeding a threshold concentration $C_{o}$. If excluded volumes of these suspended particles overlap, the total volume available for the non-interacting polymers increases. The newly available volume for polymers in solutions leads to an increase in entropy for the whole system; and (**b**) depletion forces arrange these rods with vertical as well as horizontal components (as indicated by the red arrows). If the rods are deflected along their axes, a constant restoring force arises, driving the system back to the equilibrium state with a maximized overlap (**black**) of excluded volumes. This figure is inspired by S. Asakura and F. Ooosawa, M. Hosek and J.X. Tang, and M. Kinoshita [35,36,37].

In a crowded environment, suspended colloidal particles experience a force, which tends to group them into clusters. This phenomenon of induced depletion forces was described originally as an arrangement of beads in a polymeric solution [34,35], but can be readily extended to suspended, rod-like particles [17,36,37,38,39,40,41]. Attractive forces between these particles are generated solely by the presence of free, non-interacting polymers (e.g., polyethylene glycol (PEG)) in the surrounding solution. In these environments, a small volume directly surrounding the colloids can be described as an excluded volume. This volume is hardly penetrated by polymers of the solution (Figure 2a). If a polymer enters this region, its degrees of motion will be confined due to the vicinity to the colloidal particle. A confinement of motion would lead to an entropy decrease and is thus unfavorable [34,35].

By increasing the polymer concentration, a grouping effect of the suspended colloids appears, resulting in an overlap of these excluded volumes. Accordingly, the total volume accessible for non-interacting polymers is increased leading to an entropy increase in the system (Figure 2a). Bundling, for instance, of actin filaments due to the presence of an inert polymer has been described previously [36]. The authors describe that the inert polymer PEG induces a bundling phenomenon in actin filament solutions when its concentration exceeds a critical onset value $C_{o}$ (Figure 1b). Over a limited range of PEG’s molecular weight and ionic strength, $C_{o}$ can be expressed as a function of these two variables. The process is reversible, but hysteresis is also observed in the dissolution of bundles, with ionic strength having a large influence. Additional actin filaments are able to join previously formed bundles, but PEG polymers are not incorporated into the actin structures [36]. In this picture, actin takes the role of the colloidal, rod-like particle, because PEG is much smaller and acts as the depleting agent. Bundling of rod-like particles does not only involve vertical clustering (Figure 2a) but also an axial arrangement (Figure 2b). Thus, if the ends of two filaments are shifted along the axis at the onset of the bundling process, the filaments slide along each other until the maximal overlap of their excluded volumes is reached. In turn, these filaments can be deflected from their equilibrium position by externally induced axial sliding since they are not crosslinked. Theoretical approaches have included these possibilities, revealing that two axially deflected rods in a crowded environment tend to restore the energetic minimum by a sliding motion. Exerted restoring forces have found to be of linear shape since the energy gain per unit length is constant [37,38,39]. This means that each step towards the equilibrated state contributes with the same amount of free energy when assuming a constant inter-filament spacing. Recently, these predictions have been supported by experimental findings. A system comprised of microtubules (relatively stiff biopolymers) and the depletant PEG revealed a constant restoring force when microtubules were attracted along their axes. Addition of a third rod showed that forces arise due to a pairwise additivity of the depletion interaction [40]. However, if depletion forces become very large due to high molecular crowding, the inter-filament spacing becomes smaller and smaller. In this case, electrostatic repulsion might not be sufficient to properly separate the filaments anymore and they experience frictional forces, which can effectively stall the sliding motion [41].

In general, molecular crowding effects represent a fundamental physical interaction which cannot be switched off even in active cellular systems. The cytoplasm itself is a densely crowded environment filled with macromolecules (up to 40%) [42]. Thus, sliding motions between filaments can be induced solely by depletion forces, a process which does not rely on active transport. In this regard, the depletion induced sliding or contraction of filaments can be considered a complementary or even competing process with respect to the active, dissipative cellular processes.

### 2.2. Counterion Condensation

Commonly studied biopolymers are polyelectrolytes, i.e., they carry a surface net charge. Cytoskeletal polymers like actin, microtubules, and intermediate filaments are strong polyelectrolytes, whereas extracellular proteins like collagen and fibrin exhibit low surface charges [43]. Bundling in the presence of (mostly multivalent) counterions has been described for various polymer-counterion combinations [43] and theoretical models describing the origin of attractive forces between like-charged molecules mostly refer to polyelectrolyte rods [34,44,45,46,47,48].

Although actin filaments are semiflexible, this approximation leads to valid results since the effective persistence length of a charged polymer in solution is increased due to monomer–monomer repulsion [49,50,51]. In contrast to the description of bundles formed by chemical crosslinkers or depletion interactions, forces mediated by charge fluctuations cannot be added pairwise [47,48]. However, we note that there has been some debate about this topic [52]. Calculations usually involve the “Manning criterion”, which states that mobile ions in solution condensate on oppositely charged rods when $\xi \left|z\right|>1$ [53]. In this formulation, *ξ* is the Manning parameter giving the ratio of characteristic electrostatic repulsion to thermal energy [45,53] and *z* is the ion valency. Consequentially, the charge per unit length on the (previously highly charged polyelectrolyte) rods decreases dramatically and electrostatic repulsion between them disappears for sufficiently small distances [45].

Ray and Manning have explored the complex interplay between free energy contributions of two polyelectrolytes with condensed counterions [44]. In the frame of their model, they considered the free energy of electrostatic repulsion between charge sites on the same rod, the free energy associated with repulsion between the two rods, and the energy contribution from transferring a counterion from the solution into the condensed state. Note that in the condensed state, ions are not fixed in their position, but can move freely within the condensation region. Generally, the origin of attractions is not the increased number of condensed counterions when the distance between rods becomes smaller [44]. Instead, the size of the condensation volume is the determining quantity. If the rods approach each other (mathematically from infinity), the condensation volume is initially slightly decreased yielding repulsive interactions at large distances due to a decrease in the translational entropy of the condensed counterions. By decreasing the rods’ distance further, the condensation volumes interpenetrate, which increases the translational entropy of the condensed counterions, thus leading to an attractive interaction. When compacting the rods even closer, the condensation volume shrinks below a critical threshold resulting in a repulsion for short inter-rod distances.

Another analytical description, supported by Brownian-dynamics simulations, was given for two stiff polyecletrolyte rods where all counterions are condensed [45]. Attractive interactions were found to appear at higher counterion valency. Ha and Liu assumed that not all counterions should be condensed but that free and condensed counterions are freely exchangeable [46,47]. The calculation of the interplay between two like-charged rods in a solution of counterions resulted in an interaction consisting of two parts: a repulsive term since not all counterions are in a condensed state and an attractive term due to charge fluctuations along their contours [46]. As a consequence, the number of condensed ions and the charge fluctuations between the rods increase upon decreasing the inter-rod distance. Taking the non-pairwise additivity of counterion-mediated interactions into account, the theory can be extended to describe bundles featuring more than two polyelectrolyte rods. However, in this extension the emanating attraction does not only depend on the Manning parameter *ξ*, but also on the separation between condensed ions on one rod [45,47].

Even though some of the aforementioned studies do not only involve analytical calculations but also computer simulations capturing the effects of counterion condensation [44,45,48], we would like to point to a number of publications approaching this topic via simulations [54,55,56,57].

### 2.3. Crosslinkers

Crosslinkers can be classified into physical and chemical crosslinkers. Physical crosslinkers such as van der Waals forces, depletion interactions, or certain crosslinking molecules are reversible and can be treated as an effective pairwise potential under specific conditions [58]. Chemical crosslinkers, on the other hand, are irreversible and drive the system into a frozen, disordered state. Approaches applying gelation theory to these anisotropic networks of macromolecules in combination with permanent crosslinkers [59,60,61,62] modeled the bundled state as a nematic gel and have been extensively reviewed by Benetatos and Jho [31].

Bundle formation in the presence of crosslinkers has been treated as phase transitions from isotropic polymer solutions to bundled structures [63,64]. A first approach treats polymers within solution as rigid rods and the formation of bundles occurs when the free energy of an isotropic network and the bundle phase becomes equal [63]. It comprises four contributions: (1) the increased configurational entropy of crosslinkers distributed along the filaments relative to the isotropic network; (2) the excluded volume repulsion of the rods; (3) the translational entropy of the bundles; and (4) the reduction in rotational entropy of the bundles relative to the isotropic phase. The competition of these energies and namely the entropy gain of crosslinkers in bundles compared to crosslinkers in isotropic networks favor bundle formation at low temperatures.

Within a second approach, polymers are treated as charged rods in the presence of inter-rod linkers [64]. Those linkers can be either chemical crosslinkers or counterions and are modeled as effective potentials. The energy potentials reigning the transition behavior from isotropic to bundled phase are the short-range attractive potential mediated by the linker and the long-range electrostatic repulsion between the charged rods. Each interaction is accounted for small and large angles between filaments. By adopting the generalized Onsager theory, the free energy per unit volume consists of (1) the translational and rotational entropy of the rods; (2) the interaction between two rods and (3) the contribution of free linkers as well as (4) an energy contribution of linkers adsorbed to isolated rods. Using this formulation of the free energy, the formation of a bundle phase can be deduced. In general, electrostatic repulsion favors large angles between filaments, but in bundles steric repulsions are minimized and linker binding is maximized (as opposed to a nematic phase where inter-filament repulsion is still strong).

In another study, a critical crosslinker concentration has been reported to be responsible for bundling and unbundling processes [58,65]. Above this critical concentration, filaments form either a single, large bundle or a number of sub-bundles, whereas below all filaments are unbound. This transition occurs from the competition of (1) the bending energy of single filaments; (2) the intra-filament crosslinker interaction energy; (3) the repulsion potential between single filaments; and (4) the attraction mediated by crosslinkers. Since the most dominant contributions are the energy gain by bundling due to the attractive potential and the loss of conformational entropy of the filaments, bundling occurs more easily if the filaments are stiffer.

The special case of actin bundles formed by crosslinking molecules such as fascin or espin was treated in a coarse-grained model [27,66]. It describes the thermodynamic transition from parallel unbound actin filaments to overtwisted bundles, where actin filaments are twisted with a –28/13 symmetry [15,27]. In the native configuration, actin filaments exhibit a helicity of –13/6: 6 rotations per 13 monomer repetitions [15]. Thus, parallel actin filaments are incommensurate for crosslinkers which preferably bind monomers on adjacent filaments minimally separated from each other. Overtwisting the filaments improves the binding geometry, but is accompanied with the cost of torsional energy. This competes with the binding energy of the crosslinkers, being the sum of the energy gain of perfectly aligned crosslinkers between monomers and the energy penalty of distorting the bonds. The transition from unbound, natively twisted filaments to fully bound, overtwisted filaments was found to be strongly sensitive on the crosslinker stiffness. Stiff crosslinkers cause a thermodynamic phase transition of second-order, whereas soft crosslinkers cause a smooth increase of bound crosslinkers and overtwist with increasing chemical potential. Under thermal fluctuations of filament orientation and crosslinker position, the thermodynamic distinction between the two bundling regimes is preserved [27]. Note that this model only accounts for bundles of infinite length and width [66], and the relationship between twist in bundles and finite bundle size is discussed in Section 6.

A recent description of bundle formation is based on parallel, pre-aligned polymers [67]. Two interaction terms determine the phase behavior: a short-range attraction that can be counterion-induced, hydrophobic or mediated by crosslinks or hydrogen bonds and a comparatively long-range repulsion such as electrostatic interaction between like-charged polyelectrolytes. The lateral density perpendicular to the nematically aligned polymers was studied for various interaction strengths and ranges as well as the tensions and lengths of the polymers. Depending on the interplay of the competing interactions, a macroscopic phase separation with infinite bundle size or a microphase separation with finite bundles occur. As reviewed by Benetatos and Jho [31], a similar mechanism leads to bundles in brushes of grafted polymers where the attachment points can move freely on a surface [68]. Due to the grafting, a repulsive interaction is not needed for likewise bundle formation.

## 3. Dynamics in Uncrosslinked Bundles—Contractions Without Molecular Motors

**Figure 3:**
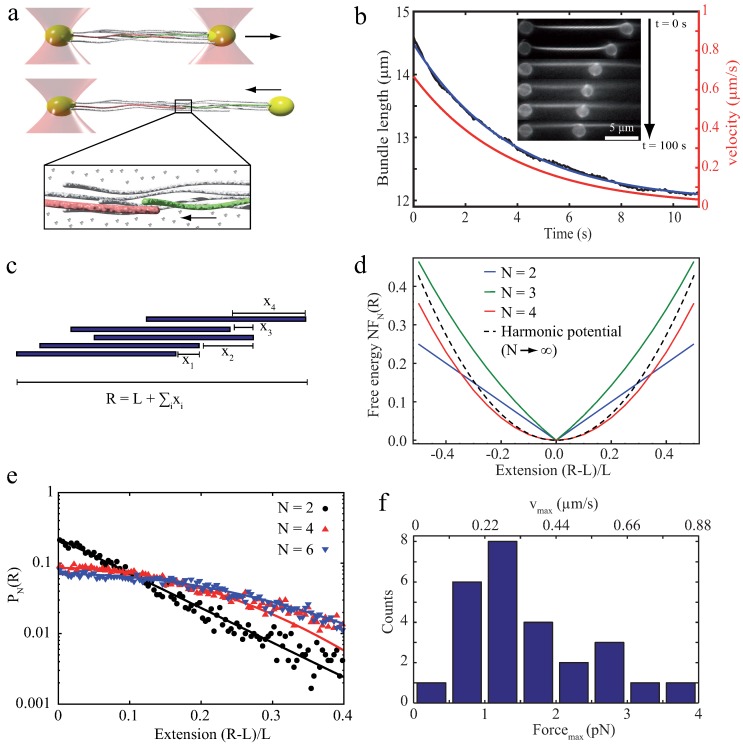
(**a**) Bundles formed by depletion forces can be deflected from their energetic minimum by inducing pulling forces via optical tweezers. After switching off the external force, bundles contract to restore the maximal overlap of excluded volumes; (**b**) recorded bundle lengths over time during contraction processes (inset) can be well described by an exponential decay function. This fitting function can be differentiated allowing an evaluation of the contraction velocity (**red** graph); (**c**) the exponential decay can be described within a mathematical model in an idealized 2D-scenario, where forces are applied at the first (i=1) and at the last filament (i=N), with *L* being the uniform filament length, *R* the length of the stretched bundle, and $x_{i}$ the displacement between filaments; (**d**) plotting the free energies $F_{N}\left(R\right)$ vs. the extension R-L shows a strong dependency on the filament number *N*. A two-filament bundle (N=2) has a linear energy landscape [37,38,39,40], but with only a few filaments (N=4) the asymptotic, harmonic form (dashed) is nearly reached [17]; (**e**) the transition from a linear to a harmonic potential shape was further confirmed by simulations. A two-filament bundle follows an exponential probability distribution as given by the Boltzmann weight. Bundles formed by six filaments yield a Gaussian probability distribution corresponding to a harmonic potential; and (**f**) measured maximal forces compare well to forces exerted by single myosin motors [2]. The figure is adapted from Schnauß et al. [17].

The initial step to understand the properties of bundles of semiflexible polymers is to investigate their properties emerging without any additional components or inner-bundle interactions. Studying effects which can be attributed to the semiflexible polymers themselves then allows to evaluate effects which are driven by additional factors such as crosslinkers or counterion condensation. Arranging filaments without these additional contributions can be achieved by crowding effects inducing depletion forces (Subsection 2.1). An example of how these bundles can be even used as a reference systems for crosslinked bundles can be found in Section 5. However, already depletion force induced bundles show a surprisingly rich repertoire of dynamical behaviors. Depletion forces cannot only arrange semiflexible polymers laterally into bundled structures but can also induce an axial sliding in a biologically relevant force regime. In the two filament case, theoretical approaches have shown that axially deflected filaments in a crowded environment slide against each other to achieve a maximized overlap of their excluded volumes (Figure 2b). The arising energy potential of this process has been reported to yield a constant force driving filaments to the equilibrium position [37,38,39]. These theoretical predictions have been experimentally verified with relatively stiff microtubules, which were grouped by the depletion agent PEG [40,69]. Using an optical tweezers setup, Hilitski et al. report that the attraction force along the axis of two microtubules is indeed constant. Upon addition of a third filament, they further revealed that arising forces are pairwise additive [40]. However, if induced depletion forces become very large due to a high molecular crowding, the inter-filament spacing becomes small. At a certain threshold concentration, electrostatic repulsion of the like-wise charged filaments might not be sufficient to properly separate them anymore. Consequently, they experience frictional forces, which can effectively stall the sliding motion [41]. However, scaling up pairwise depletion force induced filament contractions to a multi-filament scale reveals completely different dynamics [17]. To probe these dynamics, bundles of actin filaments were formed by the depletant methyl cellulose and resulting effects were only induced by attractive filament–filament interactions. For contact-free manipulations via optical tweezers, actin filaments were enriched with biotin triggering the attachment of polystyrene beads via biotin-streptavidin bonds. Actin bundles were deflected from their equilibrium position by pulling forces (Figure 3a), yielding strains that exceed normal elastic deformations (up to 175% of the initial contour length). Due to actin’s rigidity, these elongations can neither be attributed to thermal fluctuations of single filaments nor to stretching of the filament backbone. Thus, the stretching process effectively pulled filaments apart. This sliding decreased the overlap of excluded volumes of filaments and, accordingly, the freely available space for polymers within the solution. After the deflecting laser was switched off, bundles started to contract, aiming to restore a maximized overlap again (Figure 3a,b) [17]. Note that crosslinking proteins would have frozen this sliding motion and thus contractions are only induced by entropic arguments.

In contrast to the two-filament case where contractions proceed with a constant velocity [37,38,39,40,41], multi-filament bundles display fundamentally different dynamics with an exponentially decreasing velocity (Figure 3b). These dynamics translate into an exponential force decay, which, in turn, implies a harmonic energy potential. Within a mathematical model, these surprising dynamics are explained as an emergent phenomenon of rod-like colloids (actin filaments) when taking pairwise interactions to a multi-filament scale. Bundles were modeled as two-dimensional arrangements of laterally stacked pairs of rigid filaments (Figure 3c), which employs the recent experimental finding that forces are pairwise additive [40]. This approach yields an arising harmonic potential in an overdamped (highly viscous) environment (Figure 3d) when the system is composed of multiple filaments. The harmonic form of the potential and the according exponential force decay are further verified by simulations, which are independent of the mathematical model (Figure 3e) [17].

In general, molecular crowding effects represent fundamental physical interactions, which cannot be switched off even in active systems such as cells. This argument is supported by the fact that the cytoplasm itself is a densely filled environment [42]. Additionally, these bundle contractions already appear at macromolecular contents which are well below the crowding in cells emphasizing the potential biological relevance of this contractile process. Kinetics and maximal forces (Figure 3f) are in a regime of active processes based on single molecular motors, but are independent of the conversion of chemical energy into mechanical work [2].

## 4. Mechanical Properties of Bundles

### 4.1. Worm-Like Chain Model

Mechanical properties of semiflexible polymers can neither be understood in the classical physical picture of flexible polymers nor rigid rods. Due to their non-vanishing backbone stiffness, they remain in an outstretched configuration while still showing strong thermal fluctuations [2]. Within the frame of the WLC model, the polymer is described as a differentiable curve $\vec{r}\left(s\right)$ and statistical properties are determined by the effective free energy [70]

(1) $$H=\frac{\kappa }{2}\int_{0}^{L}ds{\left(\frac{\partial^{2}\vec{r}\left(s\right)}{\partial s^{2}}\right)}^{2},$$

with κ being the bending stiffness and *L* the contour length of the polymer. The tangent vector $\vec{t}\left(s\right)=\partial \vec{r}\left(s\right)/\partial s$ at the arc length *s* of $\vec{r}\left(s\right)$ is used to calculate material properties of the polymer. Fluctuations at a certain temperature allow for evaluation of the stiffness of the polymer by correlating the tangent vectors along the backbone. The arising correlation function decays exponentially with a decay constant called the persistence length $L_{p}$:

$$\langle {\vec{t}}_{0}\cdot {\vec{t}}_{s}\rangle =e^{-\frac{\left|s\right|}{L_{p}}}.$$

Thus, $L_{p}$ is a quantitative measure for polymer stiffness and if $L_{p}≅L$, the polymer is considered semiflexible. However, $L_{p}$ depicts thermal fluctuations showing a temperature dependency $L_{p}∼\;\frac{1}{T}$ and cannot be considered a material constant in general. Introducing the bending stiffness, κ, eliminates the temperature dependency by multiplying the thermal energy to the persistence length: $\kappa \;=\;k_{B}TL_{p}$ [1].

The WLC can be incorporated into theoretical approaches of assemblies of filaments into networks. However, to describe filament bundles, the theory has to be extended to depict the according behaviors sufficiently.

### 4.2. Worm-Like Bundle Model

The WLB is an extension of the WLC, which is able to describe the behavior of a bundle of worm-like chains. In nature, these filament bundles are usually held together by crosslinking proteins adding their own mechanical properties to the overall structure. Coherent with the WLC, the intrinsic material parameter for the WLB is the bending stiffness $\kappa_{b}$ of the whole bundle. This bending stiffness, however, is state-dependent and arises from the interplay of the individual filament stiffness and the relative sliding motions between filaments within the bundle [29,30,71]. This interplay leads to a sensitivity of $\kappa_{b}$ to time and length scales on which a bundle is probed.

**Figure 4:**
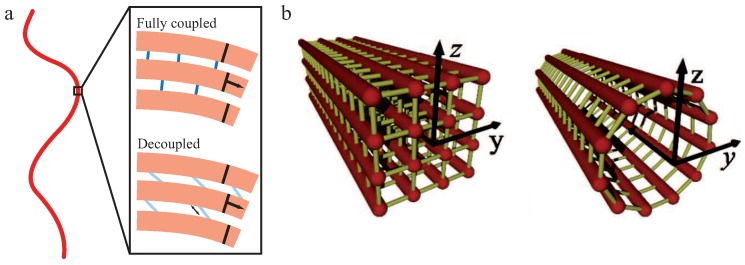
The worm-like bundle (WLB) model. (**a**) A bundle is deformed by in-plane bending, which leads to bending and stretching forces on the internal filaments (**light red**) and shearing of the crosslinkers (**blue**). These three contributions yield the overall Hamiltonian describing the behavior of the bundle. Depending on the mechanical properties of the crosslinker, two limiting cases arise: the fully coupled regime (**dark blue**—crosslinkers resist shear deformations) and the decoupled regime (**light blue**—crosslinkers deform under shear deformation). The illustration is inspired by Bathe et al. [30]; and (**b**) various bundle geometries can be considered, which yields different scaling predictions for differing bundle cross sections. This figure is adapted from Heussinger et al. [71].

Within the frame of this theory, *N* filaments of unit length *L* and bending stiffness $\kappa_{f}$ are building up a crosslinked bundle. Neighboring filaments are discretely and irreversibly crosslinked with a mean axial spacing *δ*. Additionally, crosslinkers are compliant with a shear stiffness $k_{x}$ and modeled to be inextensible and transverse to the main axis while filaments are fixed at a distance *b* (see Figure 4a). To describe the behavior of the whole bundle, three Hamiltonians are needed [29,30,71]:

(2) $$H_{\mathrm{WLB}}=H_{\mathrm{bend}}+H_{\mathrm{stretch}}+H_{\mathrm{shear}}.$$

These contributions correspond to weak bending of single filaments ($H_{\mathrm{bend}}$), filament stretching ($H_{\mathrm{stretch}}$), and crosslinker shear ($H_{\mathrm{shear}}$)

(3) $$\begin{array}{c} H_{\mathrm{bend}}=\frac{N\kappa_{f}}{2}\int_{0}^{L}ds{\left(\frac{\partial^{2}{\vec{r}}_{⊥}}{\partial s^{2}}\right)}^{2},\\ H_{\mathrm{stretch}}=Mk_{s}\delta \int_{0}^{L}ds\sum_{i=-M}^{M-1}{\left(\frac{\partial u_{i}}{\partial s}\right)}^{2},\\ H_{\mathrm{shear}}=\frac{Mk_{x}}{\delta }\int_{0}^{L}ds\sum_{i=-M}^{M-1}{\left(\Delta u_{i}+b\frac{\partial {\vec{r}}_{⊥}}{\partial s}\right)}^{2}, \end{array}$$

with *M* describing the according bundle layer, $u_{i}$ the stretching deformation of filament *i*, $k_{s}$ the filament stretching stiffness on the scale of the crosslinker spacing *δ* and $\Delta u_{i}=u_{i}-u_{i-1}$ [29].

Due to the *N*-dependency of the first term, non-trivial properties arise. A coupling parameter $\alpha \;=\;\frac{k_{x}L^{2}}{k_{s}\delta^{2}}$ can be introduced by similarity transformations allowing descriptions of the relative stiffness of a bundle’s stretching and shearing modes. Interactions of these parameters result in a new Hamiltonian

(4) $$\tilde{H}=M\int_{\tilde{s}}\left[2M{\hat{\kappa }}_{f}{\tilde{r}}_{⊥}^{{}^{\prime \prime }2}+\sum_{i=-M}^{M-1}{\tilde{u}}_{i}^{{}^{\prime }2}+\alpha \sum_{i=-M}^{M-1}{\left(\Delta u_{i}+{\tilde{r}}_{⊥}^{{}^{\prime }}\right)}^{2}\right],$$

describing, for instance, how crosslinker concentration affects the overall stiffness [29]. These interactions render a bundle a tunable structure.

Using the coupling parameter α, two limiting cases arise due to differing $\kappa_{b}$ caused by different crosslinker properties. If α→∞, crosslinkers resist shear and filaments appear as connected structures. Under deformation, the bundle’s response is dominated by filament stretching [29]. These so-called fully coupled bundles display a quadratic dependency of $\kappa_{b}$ on *N*. If α→0, filaments bend independently under deformation since crosslinkers do not resist shearing forces. These so-called decoupled bundles display a linear dependency of $\kappa_{b}$ on *N*.

In the intermediate regime, a transition from the fully coupled to the decoupled state results in a change of the scaling behavior of $\kappa_{b}$ from $N^{2}$ to *N*. Thus, the model describes two competing mechanisms with increasing *N*, crosslinker shearing and filament stretching. This cooperative effect adds new dimensions or parameters to bundled structures allowing the bundle’s response to be tuned against external deformation (see Section 5). Additionally, a combination of various crosslinkers allows to tune these properties even further. The emergence of different coupling regimes has been predicted for stereocilial bundles [72].

We note that the persistence length of bundles of semiflexible filaments has been studied in the WLB [29,30,71] but also for microtubules [71,73] as bundles of protofilaments. An important finding of the WLB is that the effective bending stiffness is not constant as for the WLC, but mode-dependent. Thus, depending on how the bundle is probed, different bending stiffnesses and, accordingly, different persistence lengths may be measured. Modeling a microtubule as an array of cylindrically arranged, fully coupled protofilaments yields a stiffness of $\kappa_{b}∼N^{3}$ [71]. This seems to contradict the aforementioned scaling $\kappa_{b}∼N^{2}$ for a fully coupled bundle, but the two results have been calculated for different bundle cross sections. A homogeneous (square lattice) bundle cross section to resemble actin bundle architecture yields $N^{2}$ and protofilaments arranged on the surface of a cylinder like in microtubules yields $N^{3}$ (see Figure 4b). The railway-track model for bundles of worm-like chains connected by harmonic springs also proposed an arclength-dependent persistence length or a renormalized bending stiffness [74].

Alternatively, microtubules can be modeled as hollow cylindrical beams that can be treated with linear elasticity theory [73]. With highly compliant inter-protofilament bonds, microtubules are able to absorb shear stresses by shifting neighboring protofilaments relatively to each other, which is in contradiction to the WLB extension for microtubules [71]. Thermal fluctuations are dominated by the high compliance of the bonds between protofilaments. Consequentially, a relationship between bundle length, i.e., microtubule length, and persistence length emerges, which agrees well with experimental results [73,75].

In the WLB, filaments are explicitly modeled as extensible polymers connected by inextensible crosslinkers with a certain shear modulus. In contrast, both the railway-track model [74] and a tension-induced bundling model [76] incorporate inextensible filaments bound together by harmonic springs. In the latter, a longitudinal tension aligns the filaments in parallel and thus induces crosslinker binding. With increasing strength and number of crosslinkers, the bundle is found to have an increased effective stiffness [76]. For infinitely strong crosslinkers, the alignment effect is even higher since transverse filament fluctuations are suppressed. Within a bundle, the extensibility of filaments and their various moduli (bending, shear, torsion) as well as the extensibility of crosslinkers and their shear modulus are needed to minimize the free energy under mechanical load and during bundle formation (see also Subsection 5.2). The mechanical response of a bundle of inextensible filaments and inextensible, infinitely stiff crosslinkers would resemble that of a stiff beam.

## 5. Transient Crosslinkers

Crosslinking proteins add an additional parameter to the properties defining the behavior of a bundle. In nature, bundles are mainly arranged by these additional components and thus understanding their role is of central importance to understand cellular processes. In general, these crosslinkers can be considered transient, i.e., they constantly bind and unbind from filaments. Experimental and theoretical approaches have revealed a rich repertoire of a bundle’s response against external stimuli.

### 5.1. Tunable Mechanical Responses of Transiently Crosslinked Actin Bundles

*α*-actinin, for instance, is a prominent example of actin crosslinking proteins forming networks as well as bundles. It has two actin binding sites and is arranged as an anti-parallel dimer [77]. Furthermore, *α*-actinin is considered a dynamic, transient crosslinker able to constantly bind and unbind from actin filaments. This allows rearrangements within actin structures as well as active or passive responses to external forces [16]. In unbound states, these crosslinkers can diffuse and subsequently bind at other positions [78].

**Figure 5:**
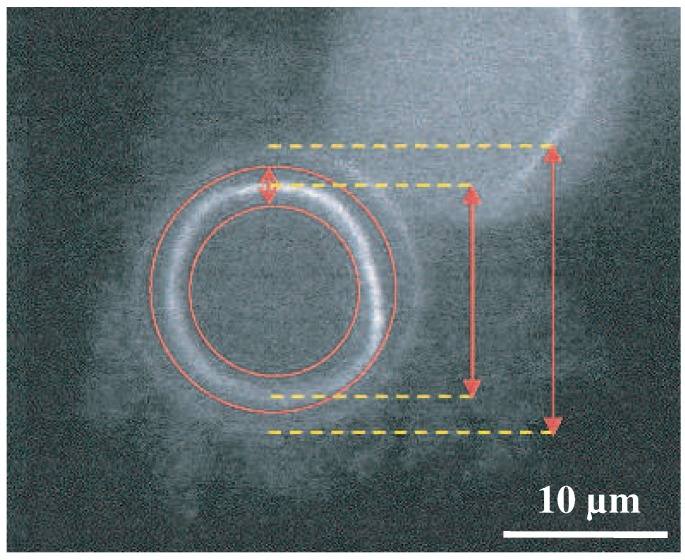
Analysis of thermal fluctuations of the actin bundle backbone (white circular shape) revealed different bending stiffnesses depending on the type of the crosslinkers. The two red circles mark the region the bundle can occupy in the time course of its thermal fluctuations. The picture is taken from Claessens et al. [13].

This transient binding is manifested in different responses against external forces when compared to permanently crosslinked bundles [13]. Thus, varying binding affinities and crosslinker dimensions are further parameters impacting a bundle’s mechanical properties. One of the first studies on these effects determined the bending stiffness of bundles enriched with differing crosslinker proteins such as fascin, plastin, or *α*-actinin [13]. By evaluating thermal fluctuations recorded with epi-fluorescence microscopy (Figure 5), the dependency of bundle size, involved concentrations, and crosslinker type to the overall bending stiffness were systematically investigated. Depending on the crosslinker type, distinct bending stiffness regimes were observed that differed by orders of magnitudes [13]. These results were a first indication of the large impact of actin accessory proteins, which has been widely underestimated before.

**Figure 6:**
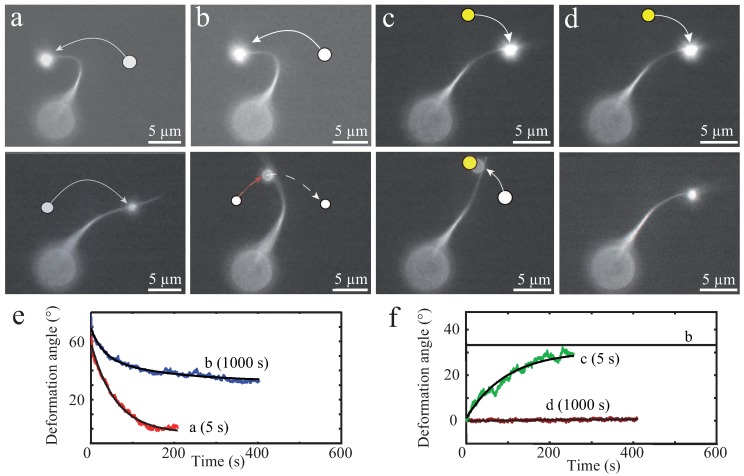
Shown are deformation experiments and according graphs (**e**,**f**) of an *α*-actinin crosslinked actin bundle and its time dependent responses to external deformations. (**a**) the bundle was deformed on a short time scale by moving the trapped bead along a predefined arc with a subsequent holding time of 5 s in the deformed configuration. For short deformations, a crosslinked bundle restored its original configuration after stress release; (**b**) deformations on a long time scale with a deformation time of 1000 s lead to dynamic rearrangements of crosslinkers causing a plastic deformation. The bundle relaxed only partially to a new equilibrium position; (**c**) the bundle was deformed to its former zero position with a holding time of 5 s. After stress release, the bundle returned to its new equilibrium position; and (**d**) this procedure was repeated with a deformation time of 1000 s, which restored the initial equilibrium position of the bundle by dynamic crosslinker rearrangements. Thus, the effect is plastically reversible with a rearranged internal structure. This figure is adapted from Strehle et al. [16].

These in vitro experiments already draw a complex picture of possible bundle responses against external stimuli. As illustrated by Strehle et al., transient crosslinking of bundled actin filaments causes time-dependent mechanical responses due to dynamic rearrangements of crosslinking proteins [16]. In this study, *α*-actinin crosslinked bundles were deformed by large bendings induced by optical tweezers. Bundles displayed elastic as well as plastic responses depending on the time scale of the deformations (Figure 6). Short deformation times of 5 s yielded a fully elastic response for crosslinked as well as uncrosslinked bundles (formed by depletion forces), which restored their original positions after stress release. Tests showed the consistency of elastic responses when deforming a bundle multiple times on short time scales ensuring that the bundles were not damaged during deformation processes [16]. However, dynamics of elastic relaxations have been found to strongly depend on the bundle structure. Visible inhomogeneities within the bundle caused an additional relaxation time resulting in a distinct relaxation profile [16].

For long deformation times of 1000 s, the two types of bundles responded differently. While uncrosslinked bundles restored their initial position similar to 5 s deformations, crosslinked bundles only relaxed partially and remained in a bent shape [16]. The plastic response can be attributed to transient crosslinkers, which can unbind, diffuse, and rebind. This structural rearrangement supported the new bundle conformation induced by external forces and could be reversed by another plastic deformation [16].

These experiments illustrate how crosslinking components can change mechanical properties of single bundles. Transient binding even facilitates dynamical rearrangements, enabling time-dependent responses against external stimuli. These binding and unbinding events can even induce further dynamic effects such as contractions as recently illustrated in a system of two microtubules crosslinked by components of the Ase1/PRC1/Map65 family [69,79,80]. We speculate that this crosslinker induced sliding can be extended to the bundle level.

### 5.2. Unbundling/Rebundling of Crosslinked Bundles

While there are a number of studies investigating the formation of crosslinked bundles, only few discuss reversible crosslinkers [58,64,65,81] and unbundling [58,65,82,83,84].

The driving mechanism for “thermal unbundling” in bundles with a sufficiently low crosslinker density are lateral fluctuations of the filaments transverse to the bundle axis [58,65]. For a given crosslinker above a critical temperature, a discontinuous unbundling transition occurs since the gain in conformational entropy of the filaments wins over the energy gain from binding due to the attractive potential mediated by the crosslinker.

Heussinger and coworkers theoretically studied unbundling driven by an external force deforming the bundle [82,83,84]. Their work was inspired by the experiments described above showing the transience of actin crosslinkers and according plastic deformation of actin bundles if the external force was applied sufficiently long [16]. In their analysis, they extended the established worm-like bundle model to account for transient crosslinkers and discussed the effect of crosslinker stiffness on unbundling. With the model applying to highly crosslinked bundels, lateral fluctuations of the filaments were neglected.

In the first step [82], the crosslinker shearing energy that also occurs in the WLB model [29,30] was modified by multiplication with an occupation parameter *n*. This variable can have the values 0 and 1, corresponding to the unbound and bound state of a crosslinker, respectively. To characterize the unbundling under bundle deformation, the average crosslinker occupation 〈n〉 was calculated. Without a deformation, crosslinkers were found to unbind (corresponding to decreasing 〈n〉) upon decreasing their binding affinity or increasing their stiffness. With increasing bundle deformation, the average crosslinker occupation decreases, eventually leading to an unbundling transition. This effect is attributed to an increasing mismatch between binding sites of neighboring filaments under load. Another parameter affecting the occupation 〈n〉 is the stiffness of the crosslinker. For stiff crosslinkers, 〈n〉 exhibits a discontinuous drop with increasing bending deformation indicating a cooperative unbinding when filaments can no longer be stretched out by the strong crosslinkers. Bundles with soft crosslinkers exhibit a smooth decrease in 〈n〉 with increasing bending. In those bundles, crosslinkers are claimed to unbind one after another.

A more detailed analysis including extensive Monte Carlo simulations was performed by Vink and Heussinger [83]. Again, the two competing energies result from the crosslinker shear with the occupation parameter and from stretching the filaments due to bending. Accordingly, filaments are more prone to stretching if bundled by stiff crosslinkers. In the case of more than two filaments bundled by stiff crosslinkers, a series of first-order transitions with increasing deformation is predicted, resulting in several sub-bundles.

In a third step, the possibility of crosslinker remodeling was considered [84], which can lead to plastic deformations of a bundle under external load [16]. The shearing energy was further modified to allow for rebinding of a crosslinker which remained attached to one filament. The second binding domain of the crosslinker can either rebind to its original binding site directly opposite on the neighboring filament or to the next right neighboring site. The latter rebinding case induces a certain mismatch between the two filaments, considered a defect in the bundle. By comparing bundles of inextensible and extensible filaments, it was found that filament stretching delayed the deformation of defects.

The effect of tensile stress on a bundle of two weakly bending WLCs connected by reversible crosslinkers has also been investigated [81]. As an extension of the tension-induced bundle model presented by von der Heydt et al. [76] and discussed in Subsection 2.3, a binary variable (comparable to the occupation parameter as explained above) was introduced to account for bound and unbound crosslinkers. Consequently, the average fraction of bound crosslinkers was analyzed for a range of tensile forces. The result displays a discontinuous phase transition from weakly bound polymers to strongly bound polymers with increasing tension. At low forces, the filaments are still capable of strong transverse fluctuations, while at higher tension suppressing these fluctuations comes not at a high entropy cost and the filaments can be bound together more tightly. The sudden crossover to a tight bundle might be interpreted as a force-stiffening mechanism [81].

## 6. Finite Bundle Size

Theories considering the bundle formation process as a phase transition from a solution of free polymers to a bundled phase predict infinite bundles [31,63,64]. However, experiments have shown that bundles exhibit a certain range of diameters [14,25]. The origin of finite bundle sizes has been attributed to a number of effects [85,86,87,88,89,90,91].

Especially for counterion induced bundles, the formation of an infinitely large bundle, spanning the whole sample, is thought to be prevented by an energy barrier impeding addition of more polymers to an already existing bundle [85,86]. This energy barrier could originate from an angle-dependent repulsion between charged bundles and polyelectrolyte rods [85]: if a rod and a bundle meet each other in a non-parallel way, the electrostatic repulsion between them is too high for further bundle growth. Thus, with an increasing bundle size, the energy barrier should grow as well [85]. Another explanation for an energy barrier hindering bundle growth are steric interactions inside counterion induced bundles [86]. Since ions have a finite size, steric interactions between them might prevent dense packing of the rods and the neutralization of charges necessary to add more rods. Additionally, during bundle growth the rods might encounter frustration also inhibiting complete charge neutralization [86].

As mentioned in Subsection 2.3, finite bundles occur in a microphase separation in a system with short-range attraction and longer-range repulsion between parallel pre-aligned polymers [67]. As long as the attractive forces are not too strong and the polymers have finite tension, the areal density of the polymers is modulated with a characteristic wavelength corresponding to bundle size [67].

**Figure 7:**
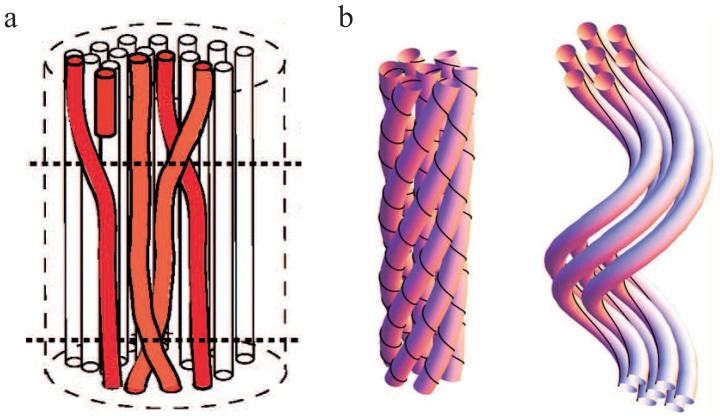
(**a**) Structural defects of the inner-bundle geometry impede the energy minimization during the bundle formation process, thus limiting the bundle size [87]; and (**b**) the molecular structure of the filaments impact the overall bundle geometry. Chiral filaments twist themselves around the bundle axis (**left**) while helical filaments cause a writhe of the bundle axis (**right**) [89]. Figures are adapted from N.S. Gov and G.M. Grason [87,89].

For neutral, achiral filaments in a bundle, structural defects have been discussed as a potential principle leading to a limited bundle size (Figure 7a) [87]. A defect inside the bundle decreases the adhesion energy and increases the elastic energy of bending and twisting the filaments, which contradicts the bundle growth. Consequentially, a minimum energy configuration arises which corresponds to a finite bundle size.

Several studies refer to chirality and twist in bundles as the main restriction factor of bundle growth [88,89,90,91]. Twist in bundles has been observed in a number of experiments [15,18,27]. Even one of the first models describing the mechanics of a bundle of two worm-like chains predicted twist in three dimensions [74]. Grason and coworkers have investigated the energy contributions for hexagonally ordered bundles of chiral and helical filaments and found that both geometrical constrictions limit bundle growth [88,89]. These results concur with the overtwisting bundle formation process [27,66], which is summarized in Subsection 2.3. Chiral filaments have to twist around the bundle axis and the energy cost of adding as well as twisting new filaments limits the bundle diameter. Helical filaments force the bundle axis itself to twist and outer filaments added to the bundle have to unbend from their intrinsic geometry (Figure 7b). The competition between the energetic cost of unbending a helical filament and the cohesive energy of the bundle leads to a finite bundle size. Whereas helical filaments are modeled with an intrinsically twisted backbone, chirality of single filaments is accounted for by a distinct crosslinker distribution on the filaments. Crosslinker sites that are helically distributed on the filaments have also been considered in an extension of the worm-like bundle model [91]. For sufficiently stiff crosslinkers, filaments inside the bundle are found to twist superhelically. The competition between the bending energy of the filaments and crosslinker induced twist determines the magnitude of twist and the equilibrium bundle width.

Monte Carlo simulations investigating the chirality in bundles also confirmed that the energetic cost of twisting filaments grows faster than the gain in adhesion energy by adding a new filament layer to a bundle [90]. Further simulations examining finite lateral bundle size have been reviewed extensively by Benetatos and Jho [31].

## 7. Bundles in Biological Systems

**Figure 8:**
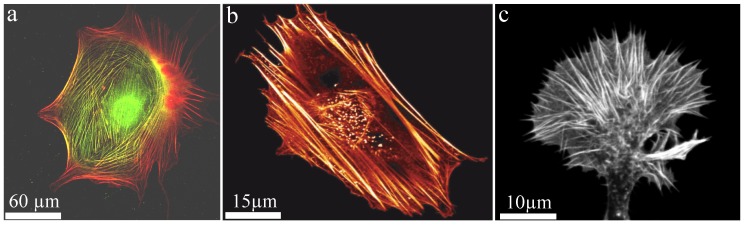
Actin bundles build up stress fibers within the cytoskeleton giving a cell structural stability. (**a**,**b**) Stress fibers entirely span throughout a stationary Balb3T3 fibroblast ((**a**) myosin labeled **green** and actin **red**; (**b**) actin **red**); (**c**) actin bundles stabilize a NG108 neuronal growth cone and according filopodia. Images were taken by Thomas Fuhs and Daniel Koch and the figure is adapted from Huber et al. 2013 [2].

Generally, polymers can be produced synthetically but also appear in a wide variety in nature. Well known biopolymers are DNA and proteins, which are crucial elements for life. In particular, semi-flexible polymers are the basis of biological compounds since they stabilize systems already at a low volume fraction. Additionally, they remain in an outstretched configuration due to their stiffness (enabling active transport) but still show Brownian fluctuations (enabling stochastic processes). In nature, semiflexible filaments such as actin are often arranged into bundled structures. One of the main cellular functions of actin bundles is the structural support of the cytoskeleton realized by so-called stress fibers. While they provide mechanical stability due to their large bending stiffness (Figure 8), they also fulfill a variety of active functions. Astonishingly, these seemingly contradictory tasks are realized by the same key component. Furthermore, filaments within a bundle are either aligned parallel or anti-parallel, where the latter enables contractile forces by myosin motors. Additional factors such as formin can drastically alter polymerization kinetics of the underlying filaments [92,93] forming an even more complex picture of actin bundle dynamics.

In cellular systems, actin bundles are found, for instance, in brush border microvilli where they support epithelial plasma membrane protrusions. These finger-like, highly regular entities are comprised of 20 to 25 crosslinked actin filaments per bundle and increase the cell surface area to enhance material exchange with the environment, e.g., in the small intestines [32,33,94]. Furthermore, these protrusions are involved in cell adhesion and mechanotransduction. Closely related are so-called stereocilia, which are also membrane protrusions of epithelial cells stabilized by actin bundles. They are typically longer than microvilli and underlying actin bundles are up to 10μm long and contain up to 900 actin filaments [94,95,96,97]. Strictly speaking, stereocilia are no real cilia since they do not contain microtubules but are solely stabilized and driven by actin bundles. These bundles are the key structure in hair cell stereocilia, which are found in arrays in the inner ear at the moveable part of mechanoreceptors [98]. They facilitate the transduction of mechanical stimuli (e.g., sound waves) into electrical signals enabling the basic principle of hearing [99].

In general, if cells need to dynamically respond to external stimuli, crosslinked actin structures are rather static and impede dynamic processes. However, dynamical, transient crosslinkers allow to dissolve actin bundles within minutes facilitating cellular dynamics such as migration. The ability to dissolve as well as to retain bundle integrity is balanced by prestress induced by molecular motors. Myosin activity counteracts bundle disintegration by applying a steady force that enables a bundle to maintain its integrity while it is also capable of rapid structural rearrangements via dynamic crosslinking [100,101].

An important biological example of the dynamic, active behavior of actin bundles are filopodia of neuronal growth cones, which are small cytoplasmic protrusions (Figure 8c). Growth cones use these filopodia to determine the motion and growth of the entire neuron and thus the development of the nerve system. To create intact neuronal structures during embryogenesis appropriately, growth cones have to analyze their environment carefully to grow along the right paths. The involved filopodia are among the most crowded cellular entities and are filled up to 40% with macromolecules [42]. Comprising bundles are formed by crosslinkers and actuate outgrowth and retraction. Fascin and fimbrin are the prominent crosslinker examples and are known as relatively stable crosslinkers, which initially oppose these dynamics [102]. However, their binding affinity can be altered by phosphorylation yielding a drastically reduced actin binding affinity [103,104]. This mechanism itself can be regulated by interactions of the cell with its environment, which potentially acts as a trigger to initiate bundle dynamics. Thus, filopodia and their underlying bundle structure can explore their environment and responses are activated by external signals.

We note that these actin bundles stretch from the tip of the growth cone to the base of the filopodia. Interestingly, they are highly contractile entities despite they are not directly contracted by active myosin motors, which are located at the interior actin cortex [105]. This conceptual idea is supported by the natural outgrowth of actin filaments within filopodia effected by filament polymerization at the tip. This leads to highly polar, parallel filaments in the bundle rendering active myosin contractions implausible.

## 8. Conclusions

This review gives a comprehensive overview of experimental and theoretical approaches describing the diverse behavior of single bundles of semiflexible polymers. These structures can be arranged through a variety of different effects allowing to test the influence of specific parameters. However, bulk measurements such as shear rheology cannot be applied since single bundles are rather anisotropic structures within solutions. Their investigations have to be performed at the mesoscopic level with rather low throughput experiments. A major limitation is that the number of filaments within a bundle is hardly detectable, which impedes direct correlations to existing theories. Additionally, the internal arrangement can be barely explored since single filaments cannot be easily visualized or according imaging techniques influence the bundle itself leading to discordant results. Due to internal structures and additional components such as crosslinkers, a complex interplay emerges leading to a variety of dynamical phenomena. Mechanical properties of these bundled structures cannot be comprehensively described within the frame of the WLC, which led to the extension known as the WLB model [29,30,71].

Generally, semiflexible polymers have the tendency to arrange in bundles, a fact which is widely employed in nature. In small entities such as cells, bundles are potentially even more important than networks since they provide mechanical stability at a much lower volume fraction. They further allow to inhomogeneously distribute mechanical properties throughout the cell body, which is a rigorous advantage for in vivo systems. Additionally, cells are very crowded entities, which inherently trigger bundle formation even without additional components such as crosslinkers or active molecular motors. Complementing cells, the extracellular matrix is also comprised of bundles, which are built up by collagen fibers [19,106,107,108]. They stabilize tissue and also affect cellular behavior, which emphasizes the biological importance of these structures even further [109,110].

We like to note that single bundles or WLB can be even further arranged into higher ordered structures such as asters. These formations of semiflexible bundles have been reported for actin [111,112,113,114,115] and microtubule [116,117,118] systems. Recently, these structures have been also documented for experiments employing DNA nanotubes [119] as a new model system for semiflexible polymers [120,121,122]. Due to structural programming, these tubes can be precisely varied allowing to tune their persistence length [122]. We speculate that this new model system will lead to additional insights for single bundles allowing, for instance, to test the dependency of the reported phenomena on the persistence lengths of the underlying polymers.

## Abbreviations

The following abbreviations are used in this manuscript:

WLC	Worm-like chain
WLB	Worm-like bundle
PEG	Polyethylene glycol
κ	Bending stiffness
$L_{p}$	Persistence length
